# Supplementary material for: Proteomic analysis of the urothelial cancer landscape
Source: Nat Commun. 2024 May 27;15:4513. doi: 10.1038/s41467-024-48096-5 (PMC11130393; doi:10.1038/s41467-024-48096-5)
Supplement: Supplementary file 1 — Supplementary Information [file 41467_2024_48096_MOESM1_ESM.pdf]

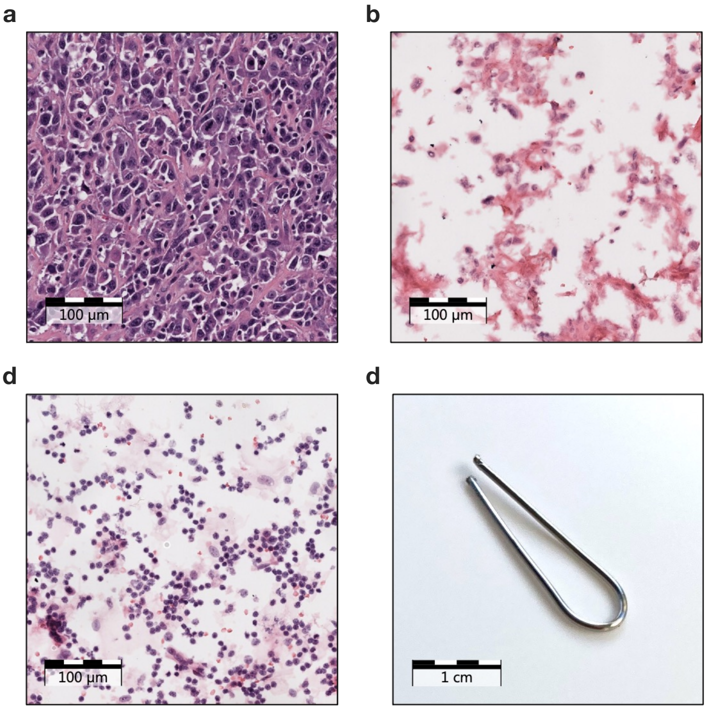


**Suppl. Fig. 1**: *Tissue dissociation by SMRD*. (A–B): Reproduction of Fig. 1B for the reader's convenience showing a hematoxylin-eosin-stained section of input tissue (A) and the result after tissue dissociation (B; stained as A); (C): In analogy to (B) but with omission of the thermal lysis and differential solubilization with intact nuclei and basophilic DNA; (D): Exemplary photograph of the self-made dissociation device.

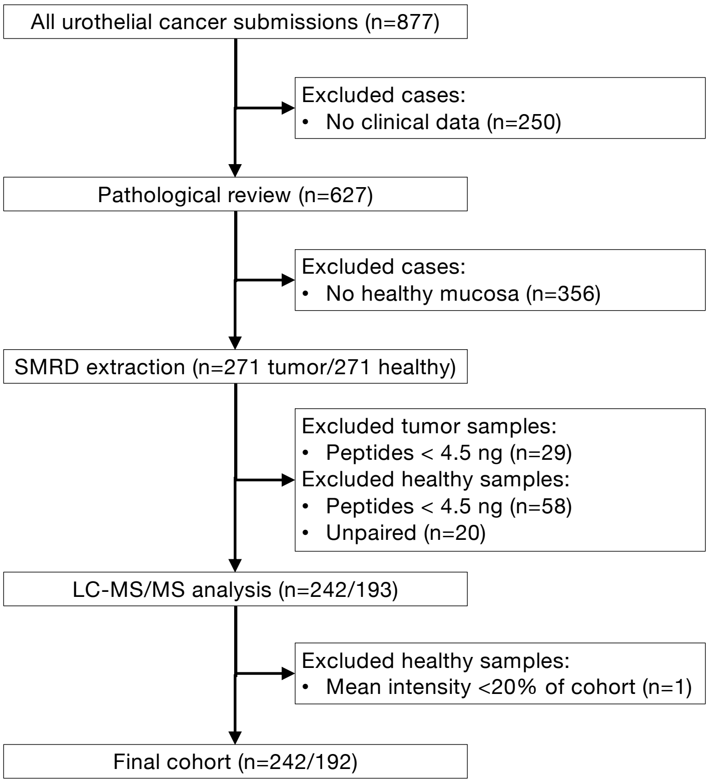


**Suppl. Fig. 2**: *CONSORT diagram for sample selection*.

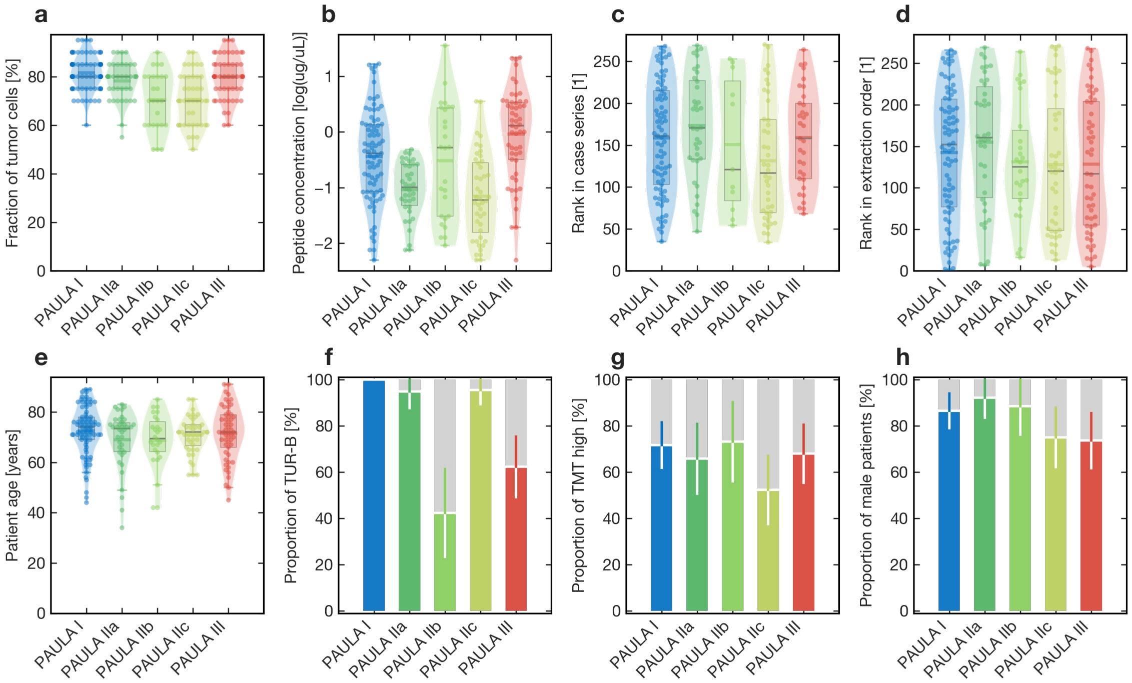


**Suppl. Fig. 3**: *Key sample and processing parameters of the PAULA cohort*. (A): The fraction of tumor cells per sample stratified by proteomic PAULA clusters (violin plot and boxplot respectively, whiskers show 95 %-interval, box is interquartile range, horizontal line is mean, horizontal bar is median); (B): The unadjusted peptide concentrations in analogy to (A); (C): Relative position in the case series (of the transurethral specimens); (D): Relative position in the order the samples were extracted; (E): Patient age; (F): The proportion of transurethral samples (bar, whiskers are the 95 % confidence interval); (G): The proportion of samples in TMT high sets; (H): The proportion of male patients.

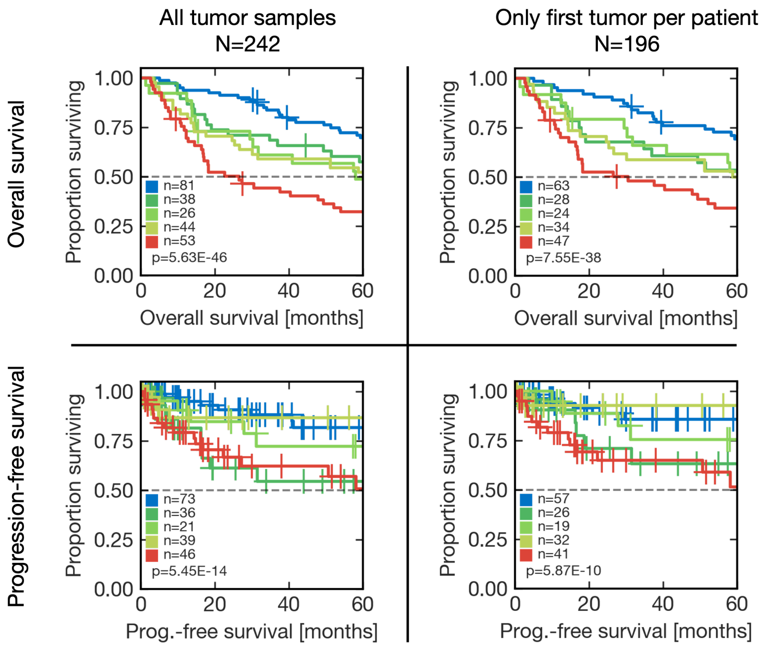


**Suppl. Fig. 4**: *Comparability of multiple metachronous samples per patient versus single samples.* Kaplan-Meier survival curves for all tumor samples (left) or only single samples per patient (initial tumor; right).

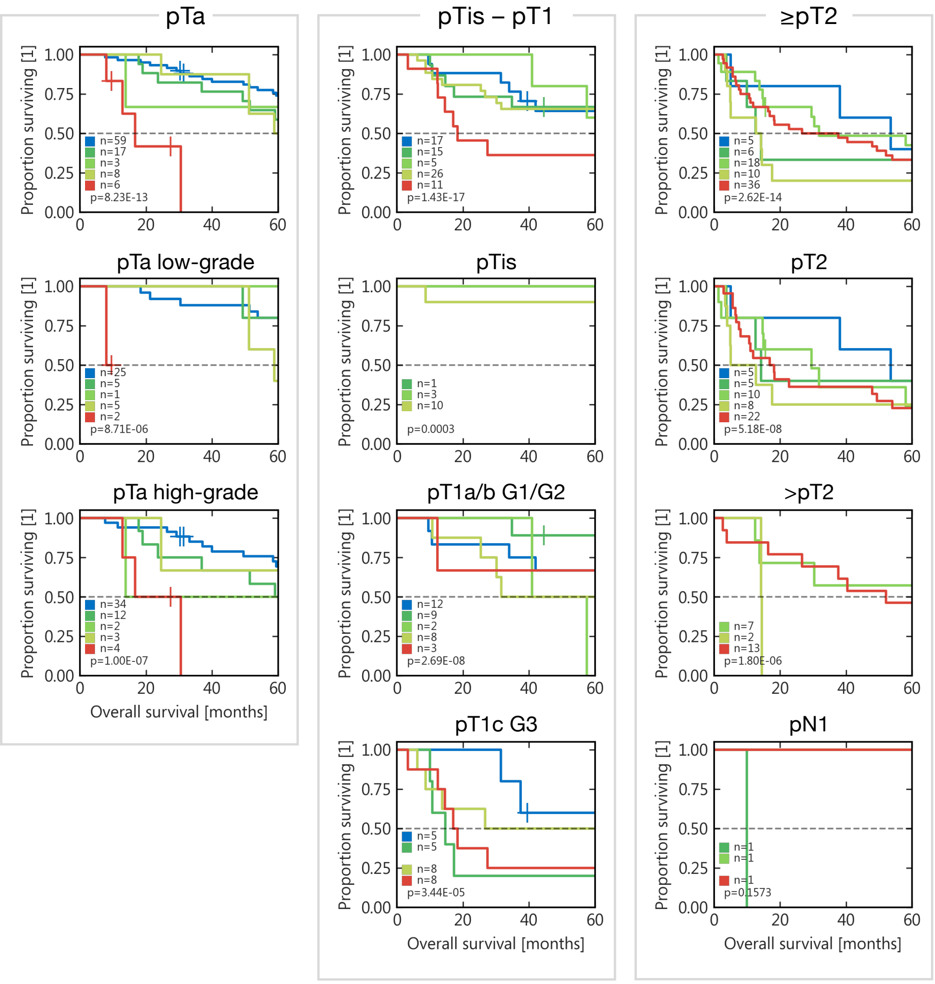


**Suppl. Fig. 5**: *Stratification of overall survival across different histopathological subgroups.*

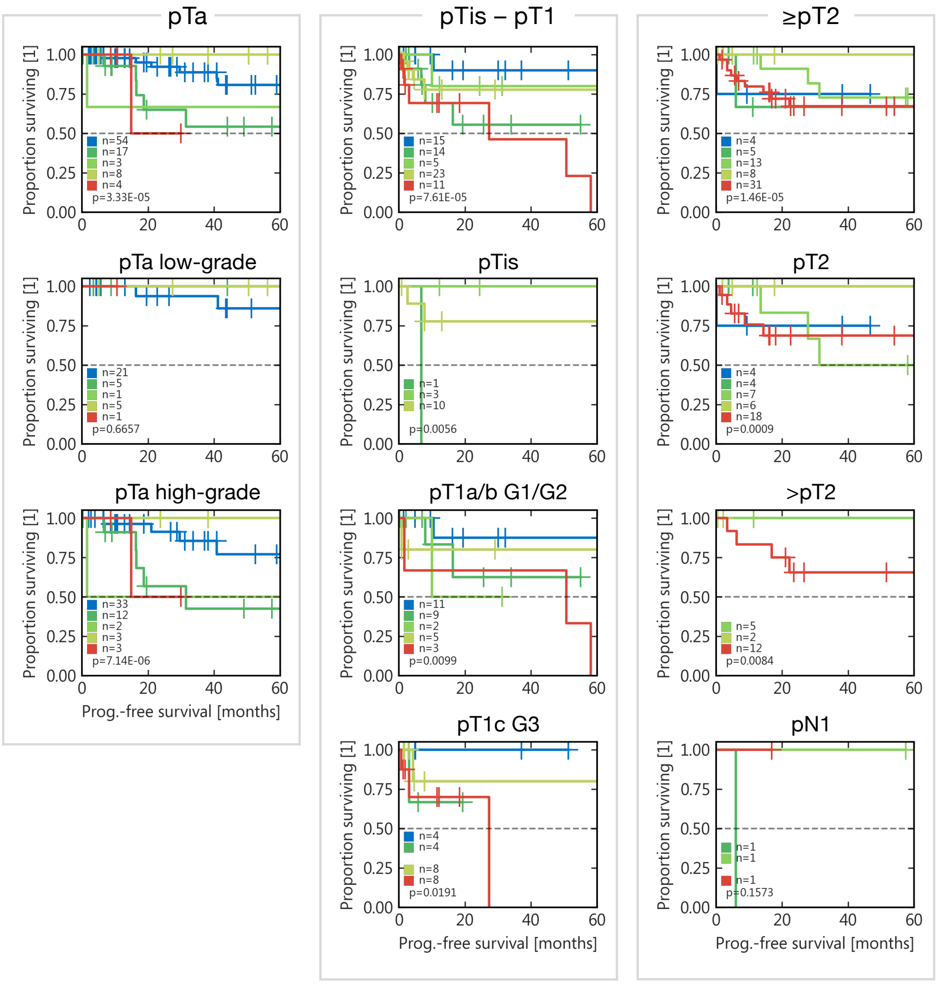


**Suppl. Fig. 6**: *Stratification of progression-free survival across different histopathological subgroups.*

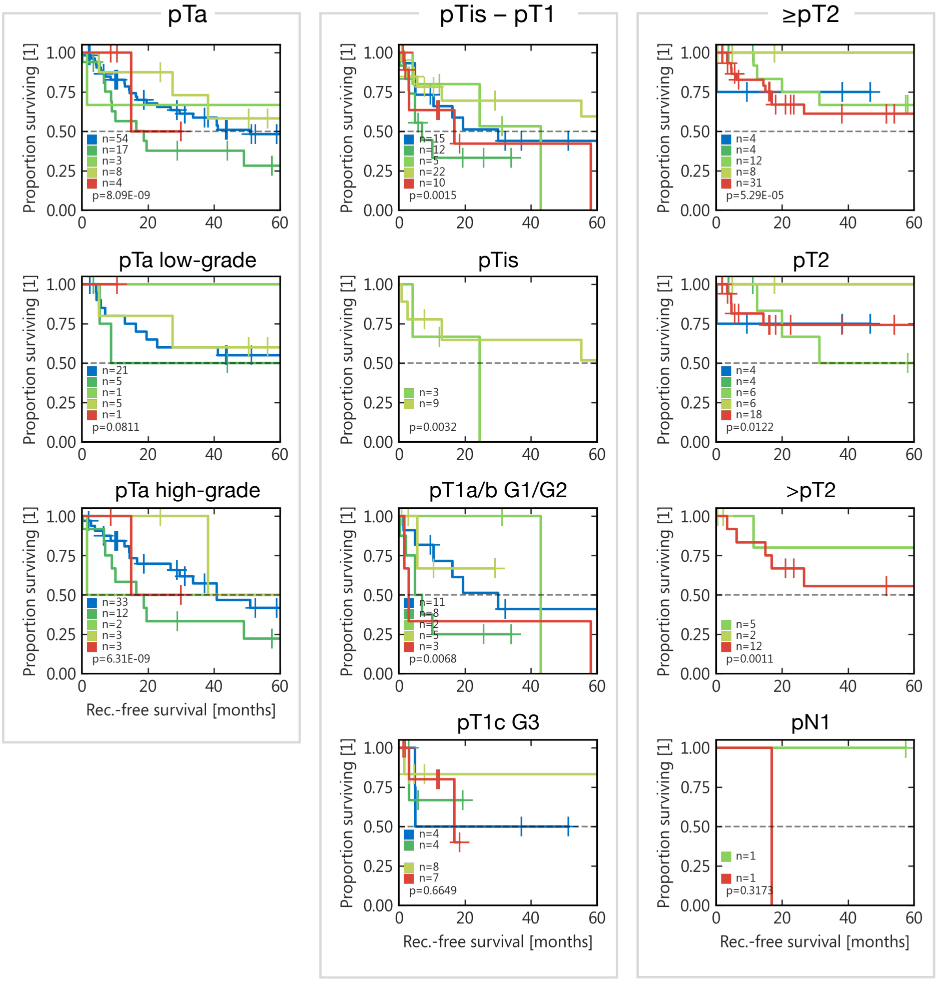


**Suppl. Fig. 7**: *Stratification of recurrence-free survival across different histopathological subgroups.*

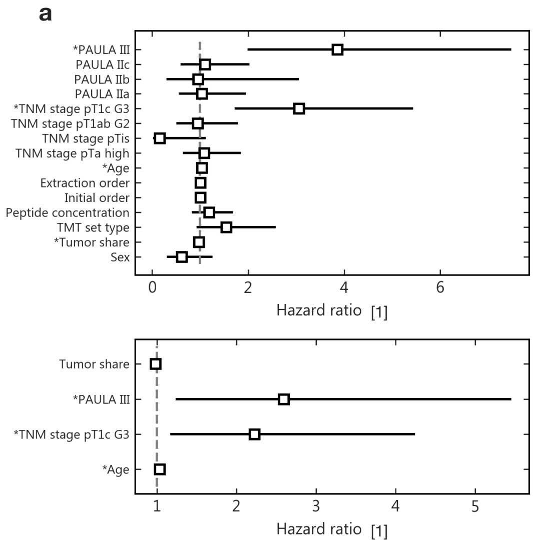


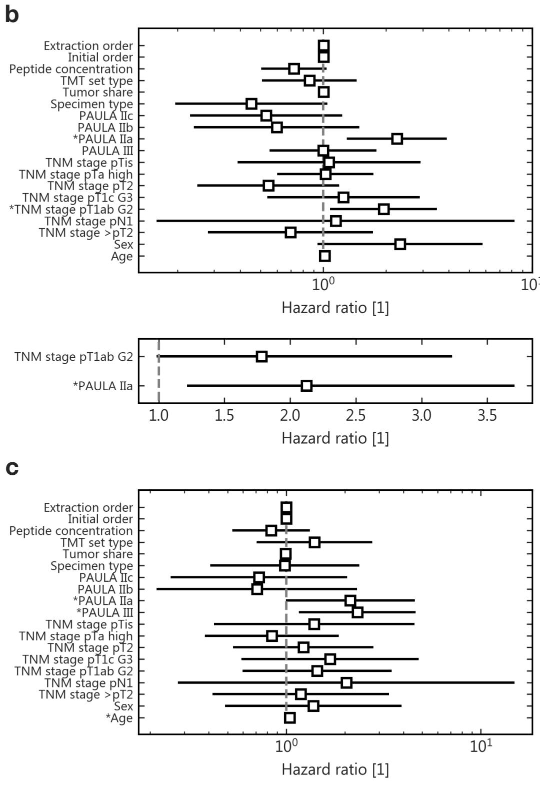


**Suppl. Fig. 8**: *Categorical Cox proportional hazards regressions for further survival data and the NMIBC subset.* *: $p \leq 0.05$; N.B.: the reference variables pTa low and PAULA I for the pTNM and PAULA categories are included as linear combination of the remaining categories; (A): Overall survival of the NMIBC samples (n=167), uni- (upper panel) and multivariate analysis (lower panel); (B): Recurrence-free survival of all samples; (C): Progression-free survival of all samples.

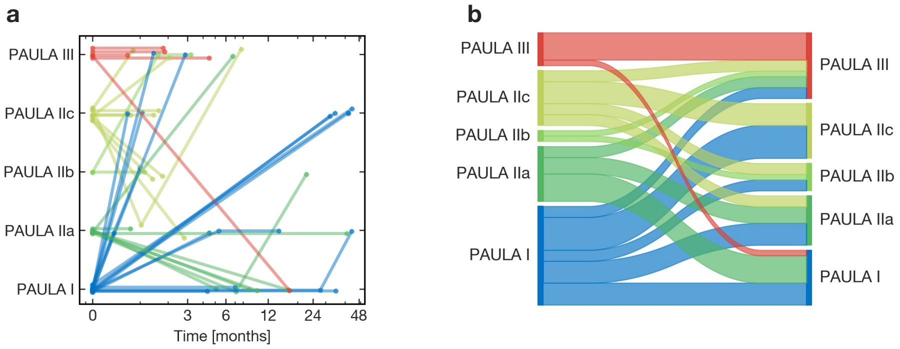


**Suppl. Fig. 9**: *Proteomic subtypes over time in the metachronous samples*. n=86 (A): Trajectories with up to three samples per patient; Color is based on the initial subtype; (B): Sankey plot of all pairwise transitions.

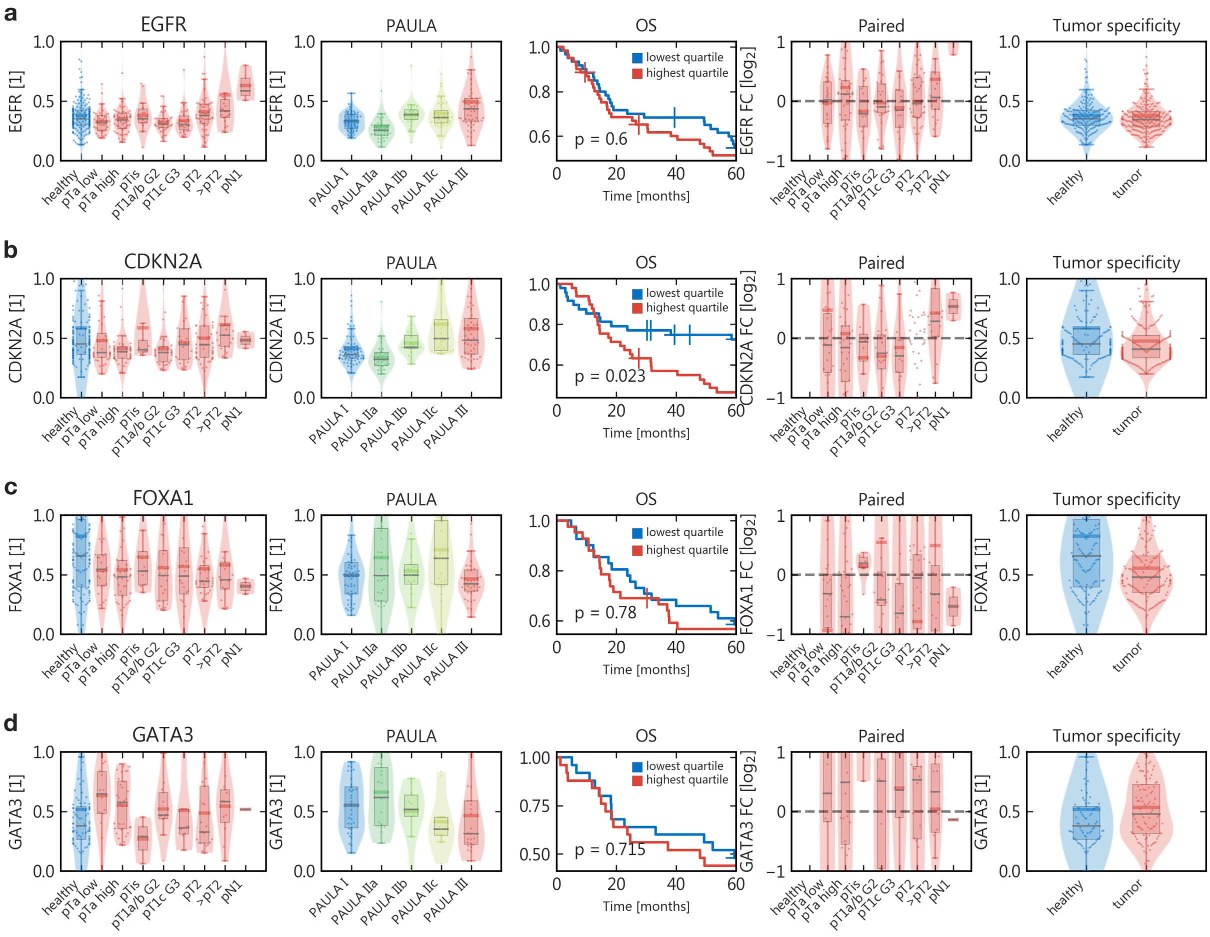


**Suppl. Fig. 10**: *Exemplary protein expression patterns.* Respective subplots from left to right: Expression across histopathological stages; Expression across PAULA subgroups; Kaplan-Meier plots for overall survival for the highest and lowest quartile; Pair-wise normalized log expression; Expression by healthy or tumor status; (A): Oncogenic receptor EGFR; n=434; (B): Genomically unstable-type marker CDKN2A (p16); n=344; (C-D): Luminal-type/negative basal-squamous-type markers FOXA1 (n=299) and GATA3 (n=179).

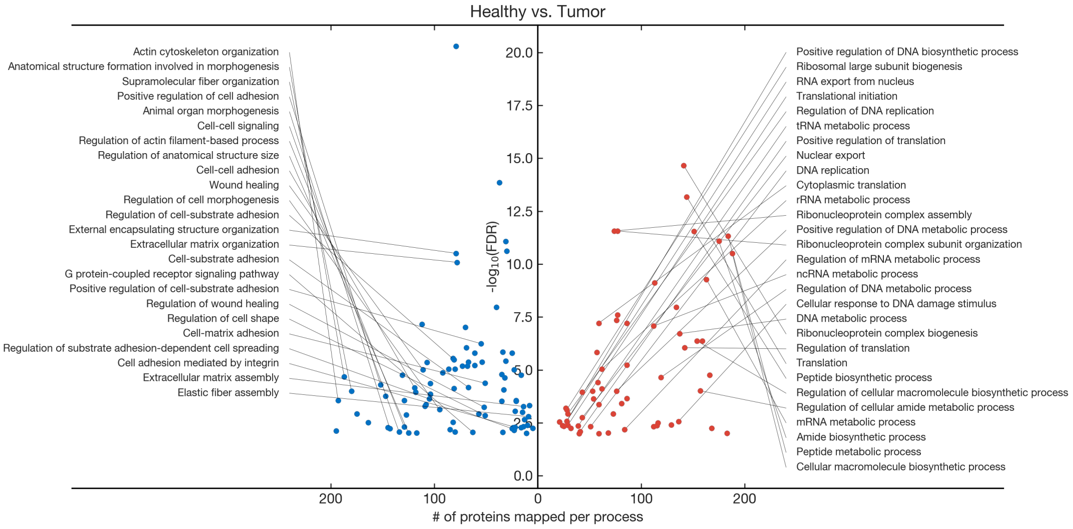


**Suppl. Fig. 11**: *Exemplary enriched biological processes of 2997 differentially expressed proteins between healthy and tumor samples.* Ranking based on the log2 fold changes.

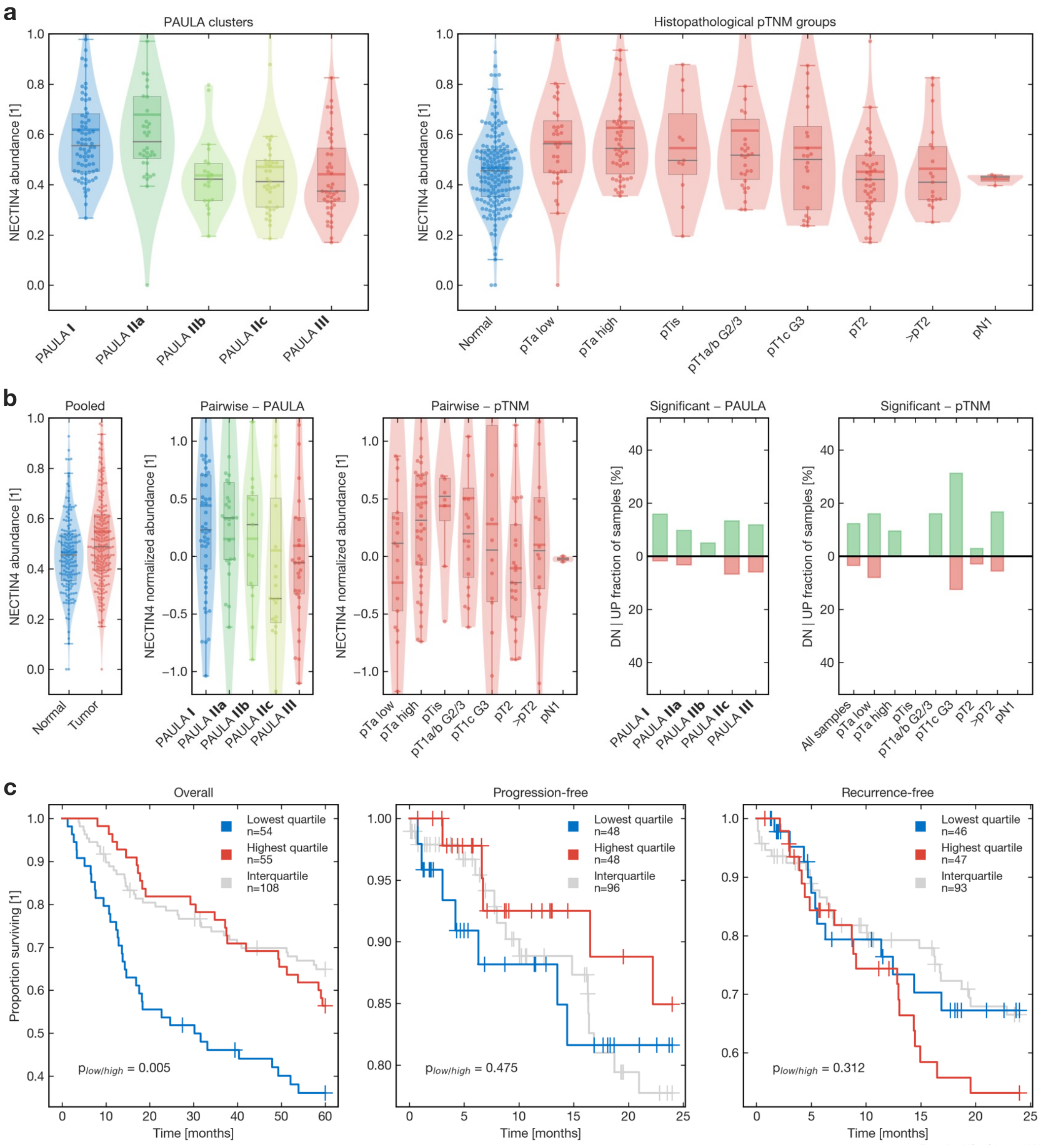


**Suppl. Fig. 12**: *NECTIN4 abundances (A), over- and underexpression rates (B) and survival stratification (C)*. n=389.

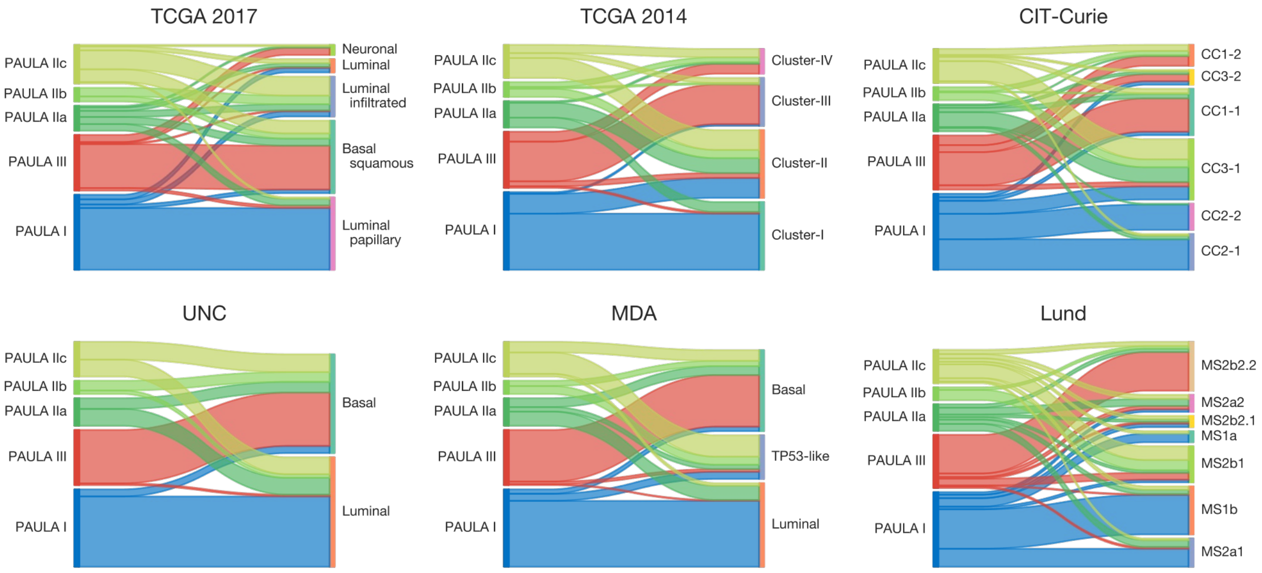


**Suppl. Fig. 13**: *Overlap with transcriptomic classifications*. Sankey plots of the proteomic (left) and respective transcriptomic clusters (right) based on all reclassified TCGA samples (n=408); Based on the supplemental data from Robertson et al. (2017).

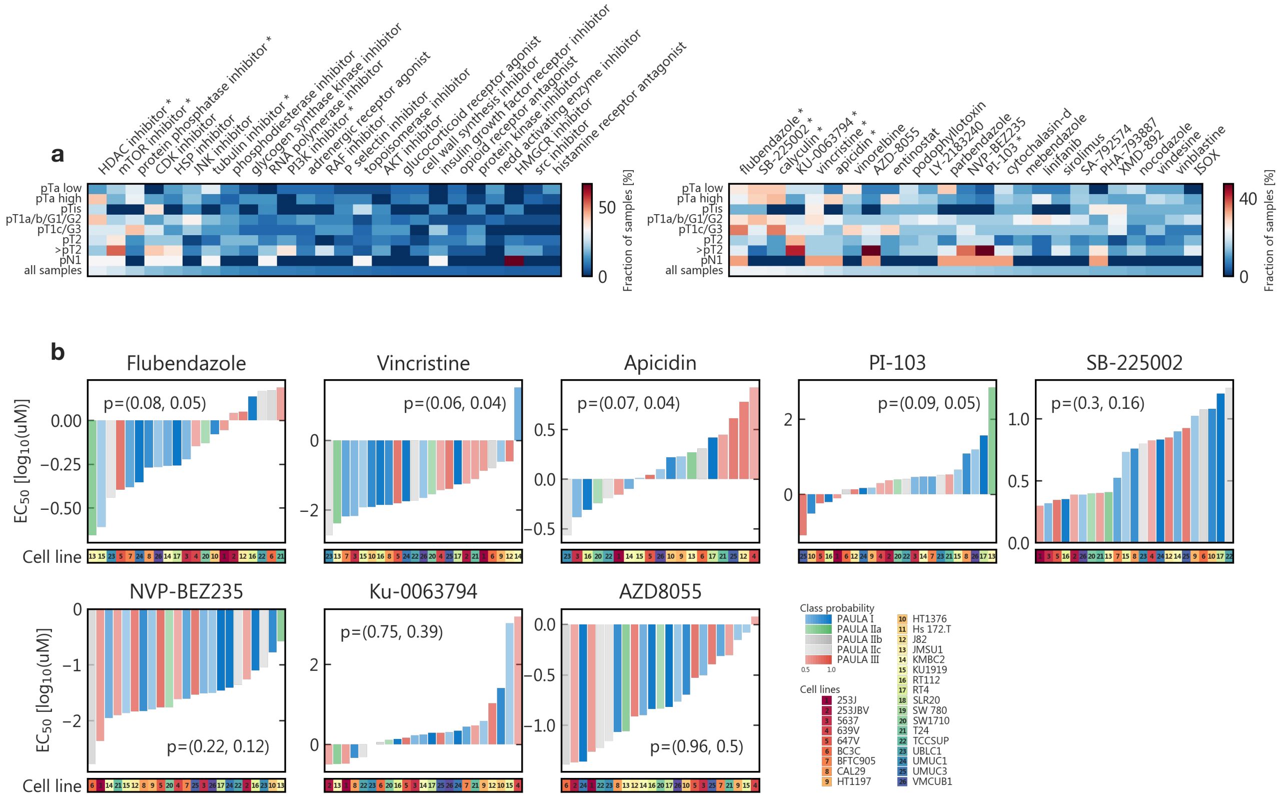


**Suppl. Fig. 14**: *Further in vitro validation of drug prediction using publicly available dose response data.* (A): In analogy to Fig. 6A for the pTNM subgroups; (B): In analogy to Fig. 6D, showing the half-maximal effect concentrations (EC50) of the respective cell lines (n=26) in ascending order; bars are medians across values from different datasets; bar base is 1 µM as indicator of sensitivity (bar downwards = likely sensitive); color is the predicted PAULA cluster based on RNA data, opacity is prediction confidence (classifier probability); p = (Kruskal-Wallis of sorted order, Mann-Whitney-U of quantitative values).

| pTNM group | n | % |
|---|---|---|
| **pTa low-grade** | 38 | 15.7 |
| **pTa high-grade** | 55 | 22.7 |
| **pTis** | 14 | 5.8 |
| **pT1a/b G1/G2** | 34 | 14.0 |
| **pT1c G3** | 26 | 10.7 |
| **pT2** | 50 | 20.7 |
| **>pT2** | 22 | 9.1 |
| **pN1** | 3 | 1.2 |

| Cell concentration | % | IQR |
|---|---|---|
| **Tumor** | 80 | [70 - 85] |
| **Healthy** | 60 | [60 - 70] |

| Peptide conc. | µg/µL | IQR |
|---|---|---|
| **Tumor** | 0.57 | [0.30 - 1.12] |
| **Healthy** | 0.28 | [0.18 - 0.48] |

| Follow-up | months | IQR |
|---|---|---|
| **Overall survival** (samples) | 92.9 | [80.7 - 113.2] |
| **Recurrence-free** (samples) | 35.5 | [9.7 - 71.9] |
| **Progression-free** (samples) | 25 | [7.1 - 60.9] |

| Samples per patient | n | % |
|---|---|---|
| **1** | 156 | 79.6 |
| **2** | 34 | 17.3 |
| **3** | 6 | 3.1 |

| Age | years | IQR |
|---|---|---|
| **All patients** | 72.7 | [66.3 - 78.2] |

| Sex | n | % |
|---|---|---|
| **Male** | 158 | 80.6 |
| **Female** | 38 | 19.4 |

| Specimen type | n | % |
|---|---|---|
| **TUR-B** | 185 | 76.4 |
| **Cystectomy** | 57 | 23.6 |

| Specimen age | years | IQR |
|---|---|---|
| **All samples** | 8.4 | [7.0 - 10.1] |

| Instillation-naive | n | % |
|---|---|---|
| **Yes** | 186 | 76.9 |
| **No** | 53 | 21.9 |
| **N/A** | 3 | 1.2 |

| Chemotherapy-naive | n | % |
|---|---|---|
| **Yes** | 235 | 97.1 |
| **No** | 4 | 1.7 |
| **N/A** | 3 | 1.2 |

| Smoker | n | % |
|---|---|---|
| **Yes** | 68 | 28.1 |
| **No** | 3 | 1.2 |
| **N/A** | 171 | 70.7 |

**Suppl. Table 1**: *Cohort characteristics*. IQR: interquartile range.

# Supplementary **Note 1**

*Objective*

Membrane proteins are difficult to identify and quantify in bottom-up proteomics due to their low abundance, low solubility and membrane and extracellular matrix adhesion (especially in formalin-fixed paraffin-embedded (FFPE) tissue). In order to optimize membrane protein enrichment and to increase analysis depth of proteomic liquid chromatography-couples tandem mass-spectrometric (LC-MS/MS) further protocols and approaches were tried.

*Enrichment by differential solubility*

Due to their lipophilic (trans-)membrane domaine and their anchoring in the plasma membrane, there are several protocols for membrane protein enrichment based on their (supposed) better solubility in a lipophilic phase. We used a commercially available kit (Biodynamics Laboratory F015, Tokyo, Japan), first for cell culture samples from T24 and HT1376 cell lines, with subsequent implementation for FFPE tissue (Fig. material S1A). While some enrichment was seen with the (high input) cell culture samples, FFPE input amounts could not realistically be scaled to balance sample loss. Furthermore, protein extracts from FFPE tissue proved thoroughly denaturated (see below) and appeared unlikely to still exhibit sufficient differential hydrophobicity.

*Enrichment by differential centrifugation*

Based on the work by e.g. Fuller et al. (2001), we tried sequential differential (ultra-)centrifugation to separate the different cellular compartments, which was unsuccessful for FFPE tissue (Fig. material S1D).

*Enrichment by liposome reconstitution*

Bearing in mind that the actual membrane domain of most membrane proteins is comparatively short, that further hydrophobic domains may well exist simultaneously, and that formalin fixation will likely crosslink membrane proteins and their surrounding lipid patches, we based our initial enrichment strategy on the selective reconstitution into artificial liposomes/vesicles. During our previous extraction optimization (Dressler et al., 2022) SDS proved central to sufficient protein extraction. Removal of SDS in turn has been used to reconstitute membrane proteins into liposomes for a variety of pharmacological and structural biological purposes (Althoff et al., 2012; Murray et al., 2014). Liposomes were created by extrusion (Merck 610000; Darmstadt, Germany) as per the manufacturer's instructions. In an initial experiment, protein extracts were directly combined in the extrusion process but were not reconstituted (data not shown). Subsequently, methyl-beta-cyclodextrin (MBCD) 250 mM was used for SDS removal and direct reconstitution (Degrip et al., 1998; Signorell et al., 2007). This yielded precipitated proteins and liposome-reconstituted proteins (Fig. material S1B). We subsequently tried several approaches to separate these components by i) variation of the reconstitution process including slow dialysis, ii) washing the liposomes with different mild detergents and solvents, iii) filtration and iv) tangential flow filtration. The latter showed highest reproducibility. While some differential efficacy was visible, sample loss was still too high for downstream LC-MS/MS (Fig. material S1C).

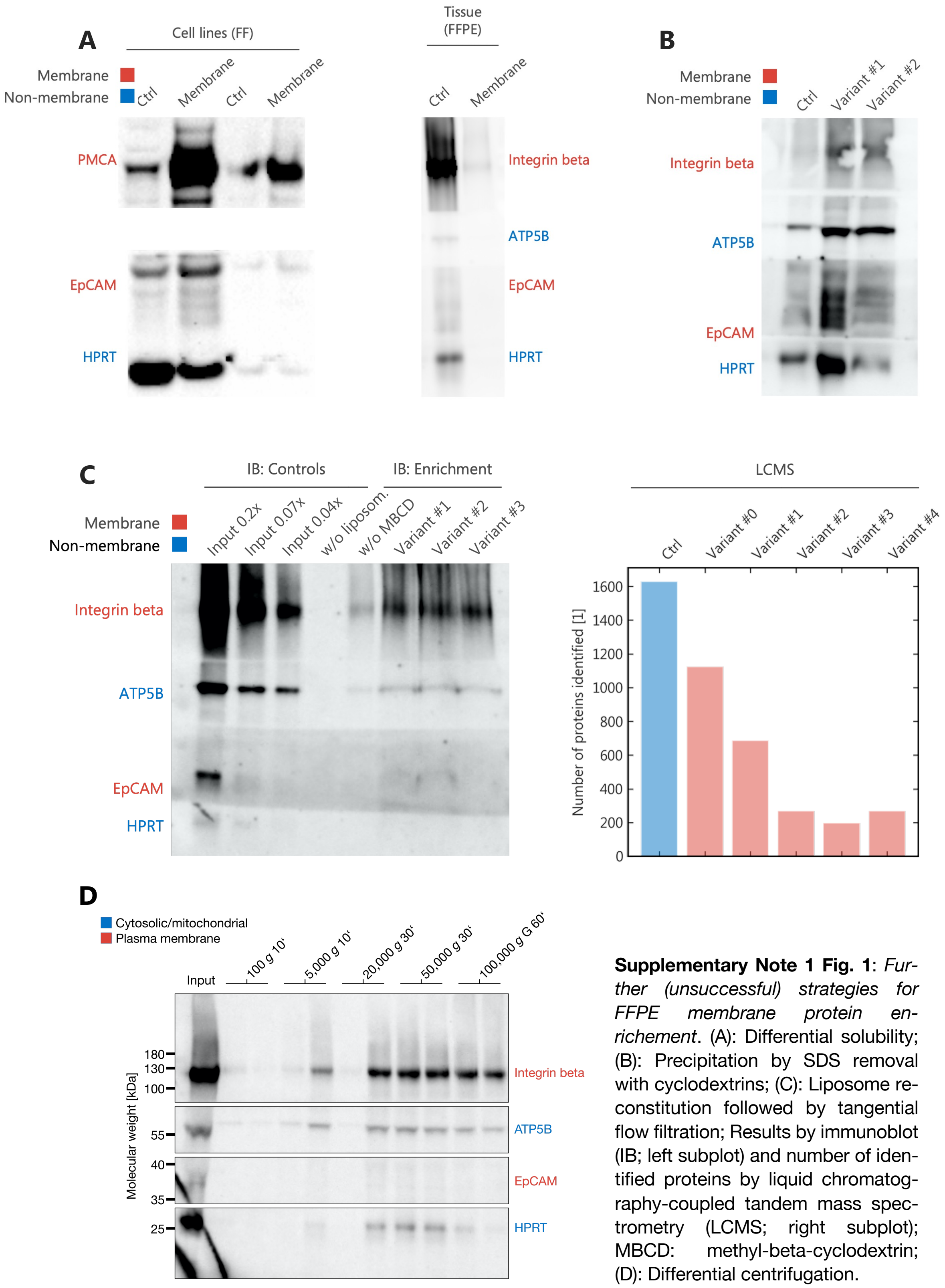


**Supplementary Note 1 Fig. 1**: *Further (unsuccessful) strategies for FFPE membrane protein enrichement*. (A): Differential solubility; (B): Precipitation by SDS removal with cyclodextrins; (C): Liposome reconstitution followed by tangential flow filtration; Results by immunoblot (IB; left subplot) and number of identified proteins by liquid chromatography-coupled tandem mass spectrometry (LCMS; right subplot); MBCD: methyl-beta-cyclodextrin; (D): Differential centrifugation.

# Supplementary **References**


Althoff, T., Davies, K.M., Schulze, S., Joos, F., and Kuhlbrandt, W. (2012). GRecon: a method for the lipid reconstitution of membrane proteins. Angew Chem Int Ed Engl *51*, 8343-8347.

Degrip, W.J., Vanoostrum, J., and Bovee-Geurts, P.H. (1998). Selective detergent-extraction from mixed detergent/lipid/protein micelles, using cyclodextrin inclusion compounds: a novel generic approach for the preparation of proteoliposomes. Biochem J *330 ( Pt 2)*, 667-674.

Dressler, F.F., Schoenfeld, J., Revyakina, O., Vogele, D., Kiefer, S., Kirfel, J., Gemoll, T., and Perner, S. (2022). Systematic evaluation and optimization of protein extraction parameters in diagnostic FFPE specimens. Clinical Proteomics *19*.

Fuller, W., Eaton, P., Medina, R.A., Bell, J., and Shattock, M.J. (2001). Differential centrifugation separates cardiac sarcolemmal and endosomal membranes from Langendorff-perfused rat hearts. Anal Biochem *293*, 216-223.

Murray, D.T., Griffin, J., and Cross, T.A. (2014). Detergent optimized membrane protein reconstitution in liposomes for solid state NMR. Biochemistry *53*, 2454-2463.

Signorell, G.A., Kaufmann, T.C., Kukulski, W., Engel, A., and Remigy, H.W. (2007). Controlled 2D crystallization of membrane proteins using methyl-beta-cyclodextrin. J Struct Biol *157*, 321-328.
