# Supplementary material for: Proteomic analysis of the urothelial cancer landscape
Source: Nat Commun. 2024 May 27;15:4513. doi: 10.1038/s41467-024-48096-5 (PMC11130393; doi:10.1038/s41467-024-48096-5)
Supplement: Supplementary file 3 — Description of Additional Supplementary Files [file 41467_2024_48096_MOESM3_ESM.pdf]

File Name: **Supplementary Data 1**
Description: *Protein abundances.*

File Name: **Supplementary Data 2**
Description: *Protein identifications and characteristics.*

File Name: **Supplementary Data 3**
Description: *Proteomic subtypes and relevant clinical data of the samples.*

File Name: **Supplementary Data 4**
Description: *Paired healthy-normalized protein abundances.*

File Name: **Supplementary Data 5**
Description: *Over- and underexpression rates for the paired healthy-normalized protein abundances.*

File Name: **Supplementary Data 6**
Description: *Differential expression analysis of the pooled healthy and tumor samples.*

File Name: **Supplementary Data 7**
Description: *Pair-wise survival comparisons for OS and PFS.*

File Name: **Supplementary Data 8**
Description: *Pair-wise statistical comparisons of the mutation frequencies between the different proteomic subtypes.*
