# Supplementary material for: Proteomic analysis of the urothelial cancer landscape
Source: Nat Commun. 2024 May 27;15:4513. doi: 10.1038/s41467-024-48096-5 (PMC11130393; doi:10.1038/s41467-024-48096-5)
Supplement: Supplementary file 12 — Reporting Summary [file 41467_2024_48096_MOESM12_ESM.pdf]

nature portfolio

Corresponding author(s): Dressler, F. F.

Last updated by author(s): Apr 7, 2024

Reporting Summary

Nature Portfolio wishes to improve the reproducibility of the work that we publish. This form provides structure for consistency and transparency in reporting. For further information on Nature Portfolio policies, see our Editorial Policies and the Editorial Policy Checklist.

Statistics

For all statistical analyses, confirm that the following items are present in the figure legend, table legend, main text, or Methods section.

n/a	Confirmed	
☐	☒	The exact sample size (n) for each experimental group/condition, given as a discrete number and unit of measurement
☐	☒	A statement on whether measurements were taken from distinct samples or whether the same sample was measured repeatedly
☐	☒	The statistical test(s) used AND whether they are one- or two-sided<br>*Only common tests should be described solely by name; describe more complex techniques in the Methods section.*
☐	☒	A description of all covariates tested
☐	☒	A description of any assumptions or corrections, such as tests of normality and adjustment for multiple comparisons
☐	☒	A full description of the statistical parameters including central tendency (e.g. means) or other basic estimates (e.g. regression coefficient) AND variation (e.g. standard deviation) or associated estimates of uncertainty (e.g. confidence intervals)
☐	☒	For null hypothesis testing, the test statistic (e.g. F, t, r) with confidence intervals, effect sizes, degrees of freedom and P value noted<br>*Give P values as exact values whenever suitable.*
☒	☐	For Bayesian analysis, information on the choice of priors and Markov chain Monte Carlo settings
☐	☒	For hierarchical and complex designs, identification of the appropriate level for tests and full reporting of outcomes
☐	☒	Estimates of effect sizes (e.g. Cohen's d, Pearson's r), indicating how they were calculated

Our web collection on statistics for biologists contains articles on many of the points above.

Software and code

Policy information about availability of computer code

Data collection	For LC-MS/MS data: ProteinPilot 5.0.2 (AB Sciex, Darmstadt, Germany); Proteome Discoverer 2.4 (Thermo Fisher Scientific, Schwerte, Germany); Proteome Discoverer 2.5 (Thermo Fisher Scientific); Mascot Server 2.5.1 (Matrix Science Ltd., UK).<br>Immunoblot: ImageLab (6.0.1; BioRad, Feldkirchen, Germany).
Data analysis	The evaluation code is available online (https://github.com/ffdre/PAULA; DOI:10.5281/zenodo.10937560), written in Python 2.7.17 and 3.9.9 and using the following packages: numpy 1.16.1, matplotlib 2.2.4, seaborn 0.9.1, pandas 0.24.2, scikit-learn 0.20.4, scipy 1.2.2, statsmodels 0.9.0, nimfa 1.16.1, lifelines 0.26.0, PROGENy 1.1.0, decoupler 1.1.0, omnipath 1.0.5, GSEApy 0.10.8. OpenCRAVAT 2.4.2 was used for mutation filtering.

For manuscripts utilizing custom algorithms or software that are central to the research but not yet described in published literature, software must be made available to editors and reviewers. We strongly encourage code deposition in a community repository (e.g. GitHub). See the Nature Portfolio guidelines for submitting code & software for further information.

Data

Policy information about availability of data

All manuscripts must include a data availability statement. This statement should provide the following information, where applicable:

- Accession codes, unique identifiers, or web links for publicly available datasets
- A description of any restrictions on data availability
- For clinical datasets or third party data, please ensure that the statement adheres to our policy

All LC-MS/MS raw data has been deposited at the PRIDE repository 131, available under proteo-meXchange.org with the identifier PXD041268 [http://proteomecentral.proteomexchange.org/cgi/GetDataset?ID= PXD041268] (cohort data), PXD041733 [http://proteomecentral.proteomexchange.org/cgi/GetDataset?ID=PXD041733] (method experiments) and PXD041873 [http://proteomecentral.proteomexchange.org/cgi/GetDataset?ID=PXD041873] (method experiments setup #1). Postprocessed data is available via an interactive web interface at www.cancerproteins.org. Source data are provided with this paper.
Several publicly available datasets were used in this study: TCGA bladder cancer RNAseq, mutation and CNV data (www.cbioportal.org/study/summary?id=blca_tcga) 9; mRNA/protein correlation data for TCGA reclassification (www.embopress.org/doi/full/10.15252/msb.20188503) 34; cell line mRNA expression data from the Cancer Cell Line Encyclopedia (www.ebi.ac.uk/gxa/experiments/E-MTAB-2770/Results) 121; corresponding protein abundances (www.cell.com/cms/10.1016/j.cell.2019.12.023/attachment/3709dedc-3a01-4e1d-ab4c-82597295c5d2/mmc2.xlsx) 123; COSMIC data for mutation filtering (via www.opencravat.org) 118; chromosome positions in GRCh38.p14 (www.ensmbl.org) 120.

Research involving human participants, their data, or biological material

Policy information about studies with human participants or human data. See also policy information about sex, gender (identity/presentation), and sexual orientation and race, ethnicity and racism.

Reporting on sex and gender	Sex was included as clinical variable in regression analyses.
Reporting on race, ethnicity, or other socially relevant groupings	There was no analysis based on race, ethnicity, or other socially relevant groupings
Population characteristics	German population, median age 73 years (interquartile range 66-78 years), 4:1 male:female ratio as there is a known increased prevalence in men (higher professional exposure to risk factors).
Recruitment	Retrospectively from a continous case series: To ensure an unbiased clinical cohort, we reviewed a continuous series of 877 UC cases including all UC submissions during a five-year period. After exclusion of external patients without clinical data, 627 cases were pathologically reviewed. 271 cases had sufficient material and matching healthy mucosa from the same case.
Ethics oversight	Ethics Committee of the University of Luebeck (19-321)

Note that full information on the approval of the study protocol must also be provided in the manuscript.

Field-specific reporting

Please select the one below that is the best fit for your research. If you are not sure, read the appropriate sections before making your selection.

[x] Life sciences [ ] Behavioural & social sciences [ ] Ecological, evolutionary & environmental sciences

For a reference copy of the document with all sections, see nature.com/documents/nr-reporting-summary-flat.pdf

Life sciences study design

All studies must disclose on these points even when the disclosure is negative.

Sample size	Sample sizes were not specifically calculated. Instead, to ensure an unbiased clinical cohort, a continuous series of 877 UC cases of the urinary bladder, including all UC submissions over a five-year period, was reviewed. After exclusion of external pa-tients without clinical data, 627 cases were reviewed pathologically. 271 cases had sufficient material and matching healthy mucosa from the same case. After SMRD processing, peptide concentrations were sufficient in 84 % of all samples. 435 samples were submitted to labelling and LC-MS/MS analysis, comprising 242 tumor samples from 196 patients and paired healthy tissue of 193 cases. 434 samples (99.7 %) demonstrated sufficient peptide intensities for quantification, with one healthy sample being excluded from further analysis, leading to n=192 sample pairs (CONSORT diagram in Supplementary Fig. 2).
Data exclusions	Please see above.
Replication	Methods setup comparison: All replicates were included in the analysis and the data shown in Fig. 1.All replicates were successfull.<br>In vitro validation: All replicates are shown in Fig. 6. All replicates were successfull.
Randomization	Not applicable, as there were no experimental groups.
Blinding	Investigators were blinded, as the study IDs were random and did not reference the original tumor group.

Reporting for specific materials, systems and methods

We require information from authors about some types of materials, experimental systems and methods used in many studies. Here, indicate whether each material, system or method listed is relevant to your study. If you are not sure if a list item applies to your research, read the appropriate section before selecting a response.

Materials & experimental systems

n/a	Involved in the study
☐	☒ Antibodies
☐	☒ Eukaryotic cell lines
☒	☐ Palaeontology and archaeology
☒	☐ Animals and other organisms
☐	☒ Clinical data
☒	☐ Dual use research of concern
☒	☐ Plants

Methods

n/a	Involved in the study
☒	☐ ChIP-seq
☒	☐ Flow cytometry
☒	☐ MRI-based neuroimaging

Antibodies

Antibodies used	For immunohistochemistry (IHC): Caldesmon (Cell Marque E89, Rocklin, USA, ready-to-use (RTU)), CD99 (Agilent 12E7, Santa Clara, USA; RTU), CD163 (Cell Marque MRQ-26; RTU), collagen IV (Cell Marque CIV22; RTU), factor XIII (Cell Marque AC-1A1; RTU), tryptase (Cell Marque G3; RTU) and osteonectin (Leica 4A4, Wetzlar, Germany; 1:10 dilution).<br>For immunoblotting (IB): anti-ITGB1 (monoclonal rabbit IgG; Cell Signaling 9699S, Leiden, Netherlands) and anti-CFL1 (monoclonal rabbit IgG; Cell Signaling Technology 5175S, Danvers, USA), secondary antibody in 1:2500 (ThermoFisher 31460).
Validation	IHC: Validated for diagnostic purposes in house with appropriate tissue controls (Caldesmon: esophagus; CD99: pancreas; CD163: lymph node; collagen IV: lung ; factor XIII: spleen; tryptase: skin; osteonectin: adrenal gland).<br>IB: Size correlation according to the literature, dynamic signal range determined.

Eukaryotic cell lines

Policy information about cell lines and Sex and Gender in Research

Cell line source(s)	Cell lines were freshly obtained from Merck (HT1376; 87032402 / RRID: CVCL_1292) or Cell Lines Service (T24; 300352 / RRID: CVCL_0554, Eppelheim, Germany) or re-typed (253J, RRID: CVCL_7935; RT4, RRID: CVCL_0036; RT112, RRID: CVCL_1670; 486P, RRID: CVCL_7940; 5637, RRID: CVCL_0126; 639V, RRID: CVCL_1048; EJ28, RRID: CVCL_5983; J82, RRID: CVCL_0359; KU1919, RRID: CVCL_1344; SW1710, RRID: CVCL_1721).
Authentication	Via commercial authentication (ATCC, STR profiling).
Mycoplasma contamination	All cell lines were tested negatively for Mycoplasma
Commonly misidentified lines (See ICLAC register)	None

Clinical data

Policy information about clinical studies

All manuscripts should comply with the ICMJEguidelines for publication of clinical research and a completedCONSORT checklist must be included with all submissions.

Clinical trial registration	Not a clinical trial
Study protocol	Not a clinical trial
Data collection	Not a clinical trial
Outcomes	Not a clinical trial
